# Supplementary material for: Vitamin D Modified DSS-Induced Colitis in Mice via STING Signaling Pathway
Source: Biology (Basel). 2025 Jun 18;14(6):715. doi: 10.3390/biology14060715 (PMC12190092; doi:10.3390/biology14060715)
Supplement: Supplementary file 1 [file biology-14-00715-s001.zip › Supplementary Materials S1: 16s-report of gut microbiota/Groups/heatmap/nogroup/non_cluster_norm_heatmap_log2.pdf]

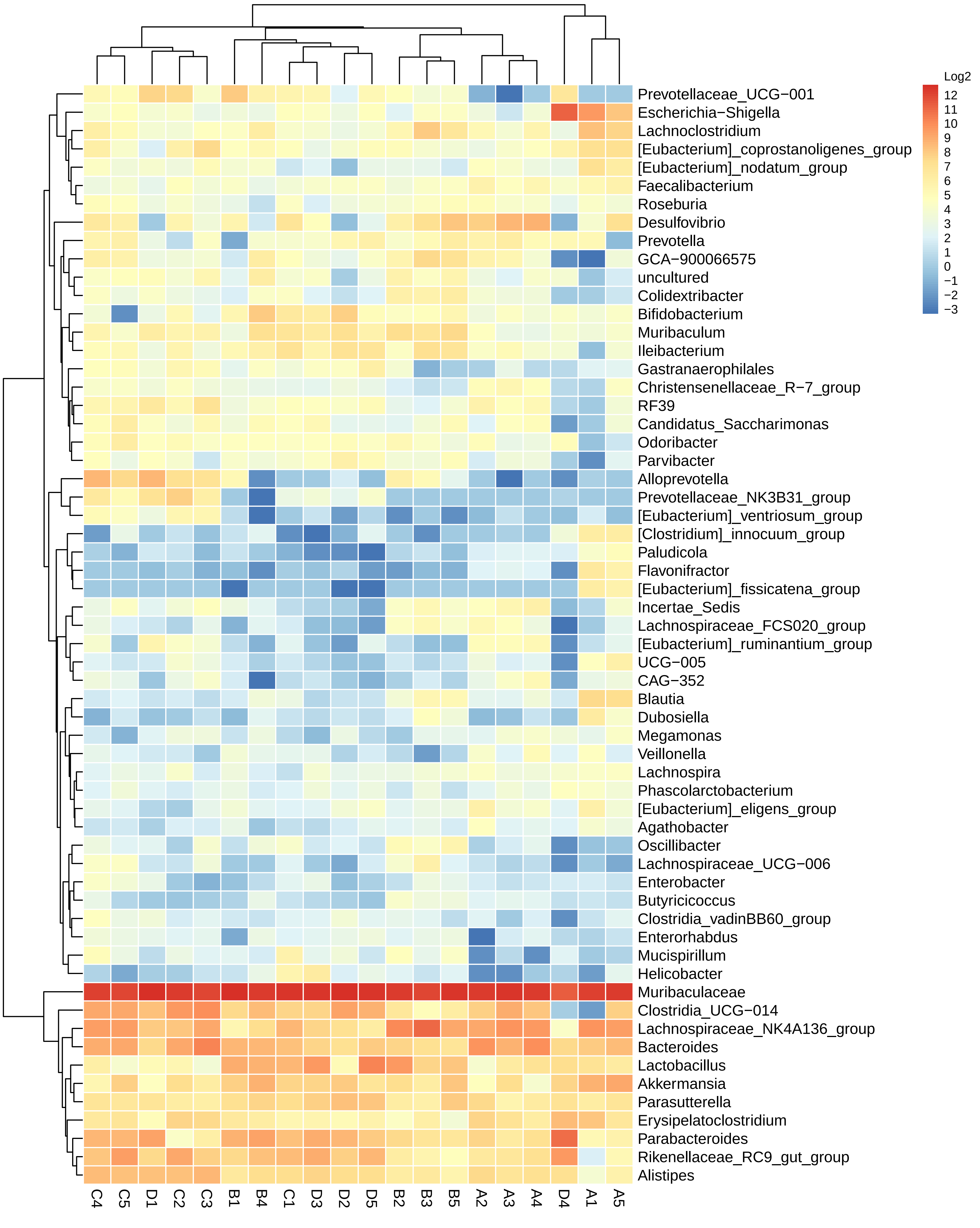
Log2
12
11
10
9
8
7
6
5
4
3
2
1
0
−1
−2
−3
Prevotellaceae_UCG−001
Escherichia−Shigella
Lachnoclostridium
[Eubacterium]_coprostanoligenes_group
[Eubacterium]_nodatum_group
Faecalibacterium
Roseburia
Desulfovibrio
Prevotella
GCA−900066575
uncultured
Colidextribacter
Bifidobacterium
Muribaculum
Ileibacterium
Gastranaerophilales
Christensenellaceae_R−7_group
RF39
Candidatus_Saccharimonas
Odoribacter
Parvibacter
Alloprevotella
Prevotellaceae_NK3B31_group
[Eubacterium]_ventriosum_group
[Clostridium]_innocuum_group
Paludicola
Flavonifractor
[Eubacterium]_fissicatena_group
Incertae_Sedis
Lachnospiraceae_FCS020_group
[Eubacterium]_ruminantium_group
UCG−005
CAG−352
Blautia
Dubosiella
Megamonas
Veillonella
Lachnospira
Phascolarctobacterium
[Eubacterium]_eligens_group
Agathobacter
Oscillibacter
Lachnospiraceae_UCG−006
Enterobacter
Butyricicoccus
Clostridia_vadinBB60_group
Enterorhabdus
Mucispirillum
Helicobacter
Muribaculaceae
Clostridia_UCG−014
Lachnospiraceae_NK4A136_group
Bacteroides
Lactobacillus
Akkermansia
Parasutterella
Erysipelatoclostridium
Parabacteroides
Rikenellaceae_RC9_gut_group
Alistipes
C4
C5
D1
C2
C3
B1
B4
C1
D3
D2
D5
B2
B3
B5
A2
A3
A4
D4
A1
A5
